# Stimulus Motion Propels Traveling Waves in Binocular Rivalry

**DOI:** 10.1371/journal.pone.0000739

**Published:** 2007-08-15

**Authors:** Tomas Knapen, Raymond van Ee, Randolph Blake

**Affiliations:** 1 Helmholtz Institute, Utrecht University, Utrecht, The Netherlands; 2 Department of Psychology, Vanderbilt University, Nashville, Tennessee, United States of America; University of Minnesota, United States of America

## Abstract

State transitions in the nervous system often take shape as traveling waves, whereby one neural state is replaced by another across space in a wave-like manner. In visual perception, transitions between the two mutually exclusive percepts that alternate when the two eyes view conflicting stimuli (binocular rivalry) may also take shape as traveling waves. The properties of these waves point to a neural substrate of binocular rivalry alternations that have the hallmark signs of lower cortical areas. In a series of experiments, we show a potent interaction between traveling waves in binocular rivalry and stimulus motion. The course of the traveling wave is biased in the motion direction of the suppressed stimulus that gains dominance by means of the wave-like transition. Thus, stimulus motion may propel the traveling wave across the stimulus to the extent that the stimulus motion dictates the traveling wave's direction completely. Using a computational model, we show that a speed-dependent asymmetry in lateral inhibitory connections between retinotopically organized and motion-sensitive neurons can explain our results. We argue that such a change in suppressive connections may play a vital role in the resolution of dynamic occlusion situations.

## Introduction

Visual perception can fluctuate over time even though the physical conditions of stimulation remain unchanged. Called bistable perception, this beguiling dissociation of physical stimulation and perceptual experience provides a potentially fruitful means for studying the neural bases of perceptual awareness, the idea being to identify changing patterns of neural activity coincident with fluctuations between perceptual states [1]. Perceptual bistability occurs in situations where the brain receives conflicting visual information about the nature of an object at a given location in the visual field. One particularly salient form of visual conflict is binocular rivalry, fluctuations in perceptual dominance between two dissimilar stimuli presented separately to the two eyes [2]. It is one important aspect of this form of bistability that provides the focus of our paper.

Upon viewing binocular rivalry for the first time, people are often struck by the appearance of the two conflicting stimuli as they experience switches in perceptual state. Transitions from suppression to dominance are not abrupt, like the changes produced when switching between television channels. Rather, rivalry transitions tend to occur in a wave-like fashion, with the previously suppressed stimulus breaking into dominance within a local area of the conflict and then spreading over the entire region. These waves of dominance during state transitions presumably reflect spatio-temporal characteristics of the neural medium promoting global perceptual dominance of one stimulus [3].

With ordinary rival targets, it is difficult to predict exactly where dominance waves will arise and in which directions they will spread. Fortunately, however, traveling waves of dominance can be controlled and measured using appropriately designed rival conditions that induce waves at specified locations and that channel their path of travel [3]. The key is to use annular rival targets together with local contrast increments to control where waves originate. Using this precise method of stimulus presentation, the properties of the waves and the medium through which they travel can be investigated. Previous experiments have implicated activity in lower cortical areas as the neural correlate of the waves, because the properties of the waves correspond to the characteristics of V1 functional connectivity [3]. This neural locus of wave propagation has been corroborated using functional imaging [4].

Thus, binocular rivalry traveling waves lend themselves to the examination of the functional properties of lower-level visual processing. We use these waves as a probe into the neural mechanisms that combine information from the two eyes under conditions of stimulus motion. In low-level stereoscopic computations, motion processing has an important role [5], [6], as evidenced by the joint encoding of motion and binocular disparity in visual cortex [7], [8], [9]. Motion also has strong effects on predominance in binocular rivalry [10], [11] and stimulus motion can interact through an interocular combination of the motion signals during binocular rivalry [12], [13]. Using counter-rotating binocular rivalry stimuli, we show that stimulus motion strongly propels traveling waves during transition phases of binocular rivalry. To explain our findings, we have developed a computational model which implements directed spatial interactions between neighboring motion detectors, a type of connectivity that may play a functional role in situations involving dynamic occlusions.

## Results

### Experiment 1 Stimulus motion propels traveling waves

**Figure 1:**
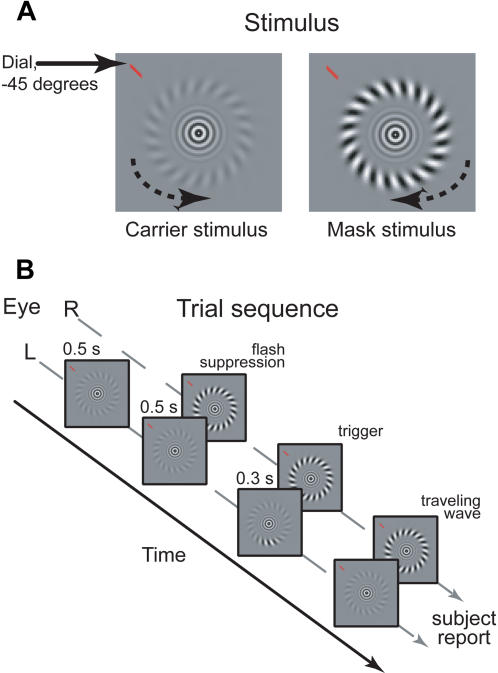
Stimuli and Trial sequence. A. Stimuli. The stimuli used in experiments 1, 2&3. Stimulus motion was varied so that speeds were equal yet opposite in both eyes to produce symmetry across the eyes. Grating orientation was also opposite in both eyes. In exp. 1, the dial position was fixed at the top of the stimulus, whereas in exp. 2, the mark was placed at different positions around the entire upper half of the stimulus. B. Trial sequence. Subjects initiated the trials by depressing the space bar and ended them by releasing the space bar. First, the lower contrast half-image (carrier grating) was shown for 500 ms. Then, the higher contrast half-image (mask grating) was projected into the other eye, causing immediate perceptual dominance of the high-contrast mask annulus due to its higher contrast and the sudden onset of presentation. After another 500 ms, a 300 ms trigger in the lower contrast carrier grating annulus was used to initiate a wave-like transition at the 6 o'clock position that propagated upward in both directions across the annular stimulus. This strict timing sequence allowed precise control over the order of subjects' perceptual state during a trial.

We investigated the interaction between stimulus motion and traveling waves in binocular rivalry using stimuli that rotated at a constant speed, which could range from slow to quite fast. Observers dichoptically viewed annular stimuli like those shown in figure 1A; the stimulus presentation sequence is illustrated in figure 1B. In short, a low-contrast grating (termed the “carrier” because it carried the wave of dominance) would be suppressed by the sudden onset of a high-contrast grating (called the “mask”). After suppression of the carrier was established, a brief, abrupt increment in the contrast of the carrier grating was introduced only at the bottom-most part of that grating; this increment triggered a duo of wave-like perceptual transitions in dominance from mask to carrier that propagated upward within the boundaries of the annular-shaped carrier, one in the clockwise direction and one in the counter-clockwise direction. The spiral angles and stimulus velocities of the two rival targets were equal in magnitude but opposite in direction for the carrier and mask; further details on the stimuli and procedures are provided in the methods section. A difference between traveling wave speed in the clockwise (CW) and counterclockwise (CCW) directions due to stimulus motion will shift the point where the traveling waves in opposite directions meet (the meeting point should fall within the arc of the wave traveling more slowly). Moreover, differences in speed will also result in a difference in arrival times of the two traveling waves at the top of the annulus.

**Figure 2:**
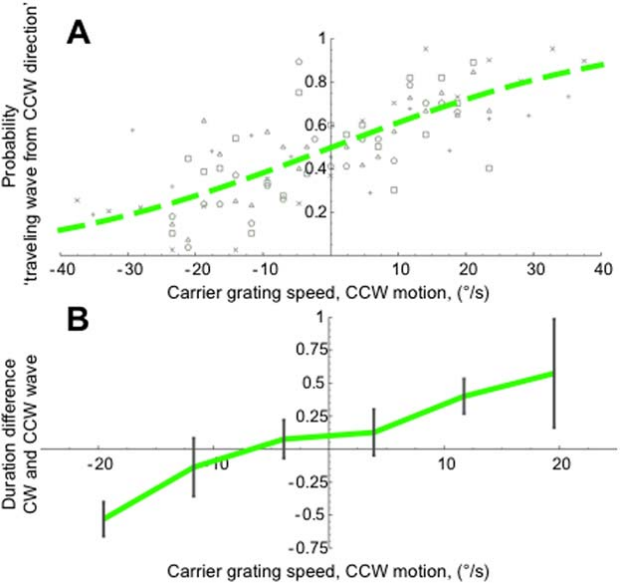
Results of Experiment 1, motion propels traveling waves. A. Experiment 1: Rates of arrival directions at different stimulus speeds depend on stimulus motion direction. Scatter plot depicts the probability that the traveling wave reached the top of the annulus from the CCW direction. Each symbol type stands for data from one of five subjects. The ordinate represent CCW probability, sampled at a certain underlying stimulus speed (abcissa), positive speeds represent carrier grating motion in the CCW direction. The green dashed line is the best-fitting cumulative Gaussian distribution. The correlation between stimulus motion and CCW traveling wave probability is highly significant (Spearman's ρ 0.77, p<<0.001). B. Differences in arrival times between CW and CCW traveling waves. Arrival times from trials of experiment 1 were binned across stimulus speeds. Stimulus motion had a significant effect on the speeds at which CW and CCW traveling waves moved (p<0.01). These data indicate that the traveling waves' tendency to arrive from a certain direction was due to a change in traveling wave speed, confirming the arrival ratio data. Data represent the mean of the difference in CW and CCW arrival time across five subjects, error bars are ±1 SEM.

Traveling waves are symmetrical when a stationary stimulus is used, meaning that they are as fast in one direction as they are in the other. Thus, the ratio of arrivals at the 12 o'clock position on the stimulus should be 50% from the CW and CCW directions for traveling waves triggered at the 6 o'clock position. Any change in this ratio would indicate an unequal change in speed of the traveling wave in the different directions. Therefore, we plotted the ratio of CCW arrivals for all 5 observers at all stimulus motion speeds in figure 2A. The data show a strong correlation between stimulus motion and the rate of CW and CCW arrival ratios, a correlation that is significant for each of the observers separately (p<0.05, *i*∈(1,5)). The green curve in figure 2A represents the best-fitting cumulative gaussian curve applied to all data. These data show that traveling waves in binocular rivalry indeed tend to move faster in the direction of the carrier's motion when compared to the opposite direction (that of the mask stimulus' motion).

If this shift in CW/CCW arrival ratio is caused by a difference in speeds between CW and CCW traveling waves, there should also be a difference between the durations of the traveling waves in CW and CCW directions. To evaluate that possibility as a control for the robustness of the difference in speeds, we measured the amount of time that elapsed between the trigger pulse and the arrival of the wave at the 12 o'clock mark. Figure 2B shows these traveling wave durations. Differences between CW and CCW traveling wave durations were binned at 8°/s-wide intervals and averaged across observers, resulting in the green line in figure 2B. The positive correlation between arrival time difference and stimulus motion is significant (Spearman's ρ 0.39, p = 0.01), as is the ratio between arrival times of traveling waves in the direction of carrier grating motion and those moving against it (p<0.05, t-test).

These influences on the duration of the traveling waves and the ratio of CW/CCW arrival compellingly demonstrate that stimulus motion significantly alters binocular rivalry traveling wave dynamics.

### Experiment 2 The magnitude of motion's influence on the traveling waves

Having found an interaction between stimulus motion and binocular rivalry traveling waves, we sought to establish a measure of its extent. At greater stimulus speeds, traveling wave arrival times encroach on the limits posed by the observer's reaction time, as the traveling waves become increasingly propelled in the direction of carrier grating motion. We therefore focused on the spatial characteristics of the ratio of CW/CCW arrivals. To allow us to increase stimulus speeds, we devised a spatial discrimination task to sample the spatial changes of traveling wave arrival probabilities due to stimulus motion. Arrival judgment position was manipulated by varying the position of the red mark across the upper half of the stimulus, providing multiple points at which the ratio of CCW and CW arrival could be assessed.

**Figure 3:**
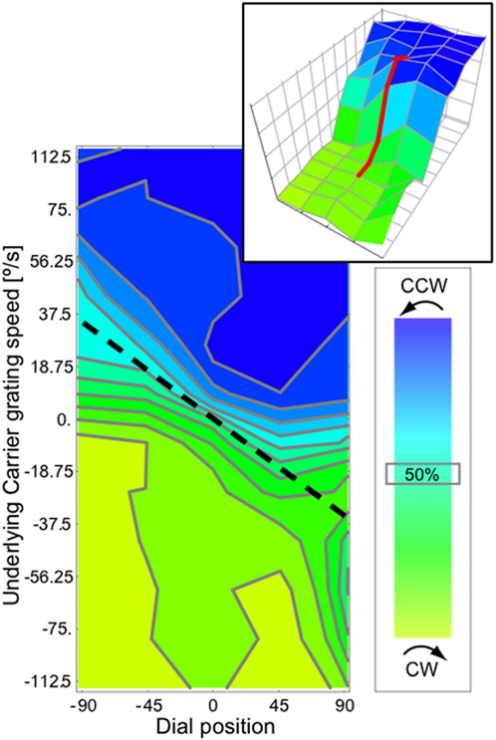
Experiment 2: Spatial shift of arrival ratios across dial positions. Underlying stimulus motion determines the position of the meeting point of the two traveling waves in both directions. Stimulus speeds in angular degrees per second are denoted along the ordinate, dial position along the abcissa. Gray lines are 9% iso-probability lines, the 3D profile is shown in the top-right inset. The black dashed line represents the line of equal μ of the best-fitting cumulative Gaussian in three dimensions. At stimulus speeds greater than approximately 75°/s in either direction the responses are dominated by the stimulus motion. In these cases, almost no reports of the traveling wave arriving in the direction opposite to the carrier grating motion occurred, even for the most extreme dial position. This means that the traveling wave moved more than three times faster in the direction of the carrier grating motion than it did in the opposite direction. Data points are the mean of 4 subjects. Inset 3D plot of the same data. The red line depicts data from the range used in experiment 1. These data mirror the data shown in figure 2A, showing that in the range of stimulus motion used in experiment 1 the results of experiment 2 show identical trends.

Results from this second experiment, shown in Figure 3, underscore the pronounced bias in traveling wave propagation due to stimulus motion, especially at higher stimulus velocities. The range of speeds at the single sampling position used in experiment 1 is represented by the red line in the 3D-inset of figure 3. The shape of this red curve strongly resembles the shape of the green curve in figure 2A (σ = 34°/s vs 30.5°/s, respectively), demonstrating that these results dovetail nicely with the results of experiment 1.

Furthermore, sampling at multiple spatial locations around the annular stimulus provides us with a robust estimate of the size of the maximal influence of stimulus motion on the traveling wave. To quantify this influence, we fit the resulting 3-dimensional curve of CCW probability (see inset figure 3) by a cumulative gaussian to estimate the angle of rotation along the z-axis (normal to the plane of figure 3), which is 40°. The data in figure 3 imply that when stimulus motion exceeds ∼±40°/s, the 50% point of the CCW probability is shifted spatially to the ±90° point, indicating that the speed of the traveling wave in the direction of the carrier grating is threefold the speed in the opposite direction. When stimulus speed is increased even further (to, say, 75°/s), this causes the motion direction of the carrier grating to fully dominate the traveling wave arrival direction.

### Model and Experiment 3 Horizontal connections between direction-selective neurons explain interaction between traveling waves and stimulus motion

**Figure 4:**
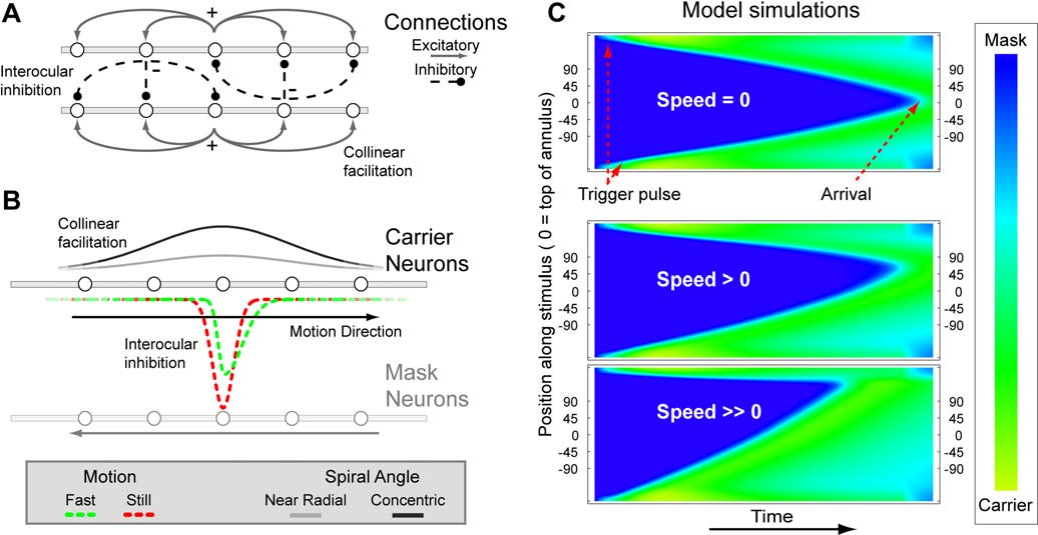
Computational model: connectivity and simulations. We adapted the model by Wilson et. al. (3) which incorporates spatially extended interocular inhibition and collinear facilitation, properties of the functional connectivity within striate cortex. A The model consists of two layers of cells, each of these layers receives input from one eye. Each cell interacts with neighboring cells in its own layer (collinear facilitation, +) and negatively interacts with retinotopically nearby cells in the opposing layer via inhibitory interneurons (−). B Illustration of the shape of excitatory (gray solid lines) and inhibitory (dashed lines) influences exerted by the layer that represents the Carrier -(*C*)- neurons. Stimulus motion causes an asymmetry in the inhibitory profile impinging on the Mask -(*M*)- neurons (green dashed curve), where a standstill stimulus causes a symmetric inhibition profile (red dashed curve). This direction-selective inhibition acts on the *M*-neurons, specifically those neurons that code for the opposite direction of motion. Thus, the increase of inhibition impinging on the *M*-neurons due to the rising activity of *C*-neurons is biased in the direction of the motion of the carrier grating. C The course of binocular rivalry traveling waves under the influence of stimulus motion, as predicted by the model. With greater stimulus speeds, the asymmetry of motion in the different directions increases and the traveling wave duration decreases. The top and bottom of the figures represent the bottom of the annular stimulus, and the sample positions used in experiment 2 are shown at the ordinate. Clearly, the point of arrival under conditions of the higher stimulus speeds lies farther than the 90° mark, meaning that the model accurately reproduces the psychophysical data. The bottom figure that represents a traveling wave under the influence of a high level of stimulus motion has a ratio between clockwise and counter-clockwise inhibition width of 4∶1.

Wilson et al (2001) developed a neural model that could account for traveling waves accompanying transitions from suppression to dominance. Their model contained excitatory and inhibitory connections among two layers of neurons representing stimulus features imaged in the separate eyes. Without revision, however, that model does not embody an effect of stimulus motion on wave speed. To examine how this effect could be explained within the context of that kind of model, we implemented the model illustrated in Figure 4.

This model is characterized by two layers of neurons with connections between neighboring cells. Each of these layers is driven by one eye's image. Both inhibitory interactions that suppress the other eye's image and excitatory connections that provide collinear facilitation extend spatially, thereby providing the lateral connections that are necessary for wave propagation (figure 4A). To produce the asymmetric course of the traveling waves revealed in our experiments, we incorporated an asymmetry in the interaction profiles between the layers of neurons that undergo mutual inhibition. This type of interaction mimics results from neurophysiological experiments which show that both excitatory and inhibitory regions in the receptive fields of V1 neurons contribute to direction selectivity [14], [15]. Anatomically, signals modeled as excitatory and inhibitory spatial interactions between neighboring neurons may travel through both horizontal [16] and feedback connections [17]. When spatially asymmetric and direction-selective inhibitory interaction profiles are incorporated, this model accurately reproduces our pattern of results (figure 4C). The prominent role for inhibitory processing follows insights from previous modeling studies [18]; [19]. The asymmetry in inhibitory interactions is due to an increase of inhibition along the path of stimulus motion. This inhibition is selectively aimed at neurons coding the direction of motion opposite to the stimulus motion direction with relatively strong speed-selectivity. The increase in inhibition in one direction is offset by a decrease in the width of the direction-selective inhibitory interactions against the path of stimulus motion. The total amount of inhibition was held constant to promote network stability. In our model, the effect of stimulus motion on inhibition is characterized by the ratio between the width of CW and CCW inhibitory interaction profiles. When both eyes receive opposite motion signals, the interocular inhibition exerted by the carrier stimulus is effectively directed at the neurons responding to the mask stimulus because of the direction-selective nature of the inhibition. When the mask stimulus is dominant during rivalry, a contrast pulse applied to the suppressed carrier grating, causes that grating to achieve local dominance and, consequently, to inhibit the mask grating more intensely along the path of the carrier motion. The result is a traveling wave of dominance that is propelled along its direction of stimulus motion. Keep in mind that the actual velocity of the carrier grating remains constant at all times - it is that grating's emergence from suppression that comprises the accelerated appearance in perception.

Figure 4C shows simulations of the model network for three stimulus motion conditions, reproducing our experimental results. The trigger pulse at the bottom of the stimulus causes a pair of traveling waves to move to the top of the stimulus. In our figure 4C the waves are plotted as moving from the top and bottom of the figure towards the horizontal midline, as top and bottom of the figure both indicate the bottom of the stimulus. When stimulus motion is 0 the waves meet at the 0° position along the circle, that is, at the top of the stimulus. However, when stimulus motion propels a traveling wave, that becomes faster in one direction than the other. For increasing speeds, this effect grows to the point where the traveling waves meet beyond the 90° mark, as occurs with the speeds greater than ∼±40°/s that occurred in our experiment 2. For the figure showing the simulation of the greatest stimulus speeds the ratio between clockwise and counter-clockwise inhibition width (an expression of the asymmetry of the inhibitory interaction profile) was 4:1.

The effect of stimulus motion is implemented as a change in inhibitory interactions, and because this change is direction-selective it should be sensitive to the relative directions and speeds of the low-level stimulus motion directions in the two eyes. This opponent direction-selective aspect of the inhibition implemented in our model allows us to make two observations: First, the direction-selectivity of inhibition explains findings in pilot experiments, in which variations of the Carrier and Mask motions that were not equal and opposite in the two eyes showed no consistent effect of stimulus motion on the traveling wave. In this case, the inhibition suppresses opponent direction-selective neurons in a limited range of speeds of preference, but these specific neurons are not stimulated due to the difference between stimulus speeds in the two eyes. Thus, the traveling wave is not influenced by these asymmetric stimulus motion conditions. Second, if the motion signals delivered to the two eyes are anti-parallel (as is the case with radial grating patterns) the effect of stimulus motion on traveling waves should be greatest, whereas increases in the spiral angle of the stimulus should attenuate motion's effect on the traveling waves. Specifically, the decrease in the effect of motion should be proportional to the cosine of the spiral angle, which is 0° for a radial grating and 90° for a pattern of concentric circles.

Furthermore, if we increase the spiral angle of the stimulus gratings, it is possible to investigate the interactions between the effects of motion and those of stimulus collinearity, which is implemented as an excitatory influence between neighboring neurons. Simulations showed that under conditions of identical angular velocities in the stimuli, increasing the excitatory gain that neighboring cells exert on one another impedes the effect of stimulus motion as represented in our model. So, both the orientation-dependence of the inhibitory asymmetry and the interaction of the inhibitory and excitatory effects would predict that the stimulus motion's effect on the traveling waves depends on spiral angle.

**Figure 5:**
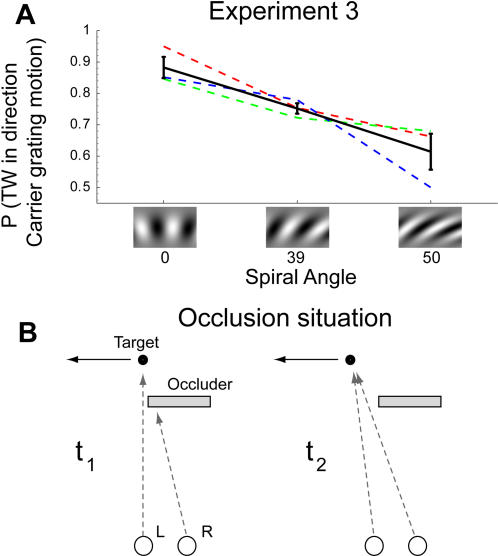
Experiment 3 and occlusion situation. A. Effect of stimulus grating collinearity on the influence of stimulus motion on the traveling wave. In our model a change in spiral angle, i.e. the collinearity of the pattern, is represented by a change in excitatory influence on neighboring neurons in the same layer, whereas this change in spiral orientation causes the effects of stimulus motion to diminish. Simulations showed that these different elements jointly act in such a way that the influence of stimulus motion is hampered. We tested this prediction directly by changing the spiral angle of both carrier and mask gratings while keeping the angular velocity of rotational motion equal at 23°/s. Data from three subjects clearly confirms the prediction of a negative effect of stimulus grating collinearity on the influence of stimulus motion. The black solid line represents the mean across subjects (colored lines), error bars are ±1 SEM. B. Diagram of the functional relevance of the implementation of asymmetric inhibition. The figures represent a top view of a binocular occlusion situation at two times, *t<t*. A moving object may be occluded in one eye (R, *t*) and visible in the other (L, *t*). Direction-selective inhibition of the right-eye neurons in the path of the motion that is visible in the left eye allows direction-selective right-eye neurons to respond earlier to the appearance of the target moving leftward at *t*.

We tested this prediction by measuring the effect of stimulus motion (indexed as the probability that the traveling wave would reach the 12 o'clock mark from the direction of the carrier grating's motion) for several spiral angle conditions, with angular rotational velocity held constant. The model predicts that as the spiral angle of the annular gratings increases, the probability that the traveling wave arrives from the direction of carrier grating motion must decrease. Data from three observers, shown in figure 5B, confirms this prediction by demonstrating a monotonic decrease of the effect of stimulus motion on the traveling wave arrival ratio as stimulus spiral angle increases for each of the observers.

## Discussion

We show that stimulus motion propels binocular rivalry traveling waves to the point that the traveling wave is dominated by stimulus motion. Binocular rivalry is usually regarded as a process that occurs at multiple neural sites in concert [2], [20], [21], however, our model explains our psychophysical results in a single-layer network using a directional inhibition asymmetry that exerts its influence on a relatively low neural level. Although this influence may be due to feedback from higher motion-sensitive areas or attentional processing [22], [23], our results do imply an important role for low-level cortical network interactions in the dynamics of binocular rivalry traveling waves, confirming previous findings using the same type of stimulus [3], [4].

Moreover, the motion-dependent asymmetry of inhibition implemented in our model is not limited to the dichoptic presentation conditions provoking binocular rivalry. In our model the same lateral interactions will be evoked during ordinary binocular viewing, which leads us to speculate that the functional significance of these asymmetric, inhibitory interactions may be importantly involved in other aspects of vision. Indeed, such network dynamics could provide valuable functionality in situations of dynamic occlusions that occur continuously in everyday life (figure 5B). When an observer views an object moving from behind an occluding surface, one eye will receive the image of at least part of that object before the other eye does. If neurons responsive to motion of that ”leading eye“ stimulus selectively inhibit neurons registering the opposite direction of motion along the projected path of the stimulus, the neurons responsive to the stimulus in the second eye will be ‘primed’ to respond to the direction of motion already present in the other eye. We envisage this type of neural wiring to play an important role in the human ability to perceive depth from interocular temporal order and motion direction [6], [24].

## Materials and Methods

### Stimuli

Stimuli were dichoptically projected by means of a mirror stereoscope. Viewing distance was 57 or 47 cm (experiment 1 and 2, resp.). Screen size was 22″, and resolution was 1600×1200 pixels, display refresh rate was 75 Hz. Screen γ was linearized. Maximum stimulus luminance was 71.1 cd m^−2^, background luminance was 35.5 cd⋅m. Stimuli were gaussian enveloped (μ = 1 dva eccentricity, σ = 0.06 dva) sinusoidal spiral grating (32 cycles, angle 30°) annuli. This spiral angle was varied in experiment 3. Luminance contrast of the carrier and mask gratings and stimulus speed range were adjusted per observer, with a mean mask contrast of 0.9, and a mean carrier contrast of 0.24. The range of stimulus motions was confined to lie between −38°/s and 38°/s in experiment 1, whereas this range was larger in experiment 2 (see figure 3). Stimulus motion was held constant at ±23°/s in experiment 3. A fixation mark consisting of concentric sinusoidal gratings with a gaussian window (σ = 0.2 dva) was always projected in the middle of the screen. To elicit a binocular rivalry transition, a gaussian enveloped (σ = 12.6 circular degrees) luminance contrast pulse of contrast 1 was projected in the carrier grating at the bottom of the stimulus. This transition boundary then moved upward across the annular grating in the form of a traveling wave until reaching a red mark projected near the stimulus as shown in figure 1A. This mark was placed at the top of the stimulus in experiment 1&3, and at varying locations (±90, ±45&0 circular degrees from the top of the stimulus) in experiment 2. Stimulus motion speed was varied in opposite directions for the carrier and mask gratings. This was done symmetrically to avoid a strong increase in the relative dominance of one stimulus half-image relative to the other due to differences in stimulus motion between the two eyes. Orientation, motion directions and which eye received a given stimulus pattern were counterbalanced and randomized during a single session.

### Task and Procedure

Observers were seated in a darkened. They initiated a trial, the sequence of which is depicted in figure 1B, by depressing the space bar. First, the low-contrast, carrier grating was displayed in one eye for 500 ms. Subsequently, the high-contrast mask grating was projected in the other eye, causing perceptual suppression of the carrier grating due to the difference in contrast and the sudden onset of the introduction of the mask grating. 500 ms after the introduction of the mask grating, a transient and local contrast pulse in the carrier grating was introduced for 300 ms to induce an transition of perceptual state in the form of a traveling wave. After the transient pulse the display continued to show the rival targets while the traveling wave moved upward around the stimulus. At the moment the traveling wave reached the mark placed near the stimulus, the observer was instructed to release the space bar. After the release of the space bar, which was timed, observers indicated whether the traveling wave arrived from the left or right side of the mark, or whether a mistrial had occurred (no traveling wave was initiated, or initial suppression was not achieved). Observers performed self-paced trials in 1 session (420 trials) for experiment, 2 sessions (320 trials each) for experiment 2 and for experiment 3, 1 session of 360 trials. In total, there were 9 different observers; 5 observers participated in the first experiment, 4 in experiment 2, and 3 in experiment 3. All observers were naive, except author TK who participated in all experiments.

### Model

We adapted the model by Wilson et. al. [3] that explains the properties of traveling waves for stationary stimuli in terms of the characteristics of low-level functional connectivity. In this model two circular arrays of neurons, each coding for a certain monocular stimulus’ percept, self-adapt slowly and inhibit one another through the activity of interneurons (figure 4). These three components, the stimulus-related (*T*), interneuronal activity (*I*) and stimulus-related activity self-adaptation (*H*) are represented by the following differential equations describing one carrier (*C_n_*) cell: 
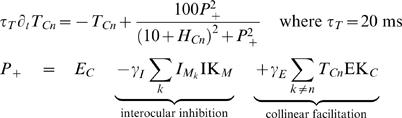
(1)

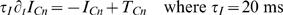
(2)

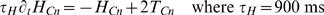
(3)Here, IK and EK are inhibitory and excitatory interaction profiles, respectively.
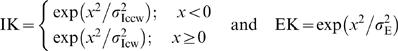
The effect of stimulus motion is modeled as increased interocular inhibition exerted by each of the monocular half-image stimuli in the direction of its own motion, pictured as the green curve in figure 4. This corresponds to asymmetric IKs due to differences in *σ* ^2^ *_Iccw_* and *σ* ^2^ *_Icw_*. The total amount of interocular inhibition was held constant regardless of the asymmetry in order to ensure network stability. Note that when *σ* ^2^ *_Iccw_* = *σ* ^2^ *_Icw_* we arrive at the model with stationary stimuli. Simulations included 136 cells for both the Mask (*M*) and Carrier (*C*) gratings, and were run with a fixed stepsize of 0.25 ms using the GNU scientific library implementation of the fourth order Runge-Kutta procedure. Values were  = 0.85, *γ_E_*∈ (0.0,0.04) for spiral patterns ranging from radial to concentric, the strenghts of the inputs of Mask and Carrier gratings  = 30 and  = 24, *σ* ^2^ *_E_* = 6.

Our model also provides clear predictions regarding the optimal durations of the phases of stimulus presentation during a single trial. In pilot experiments, for example, shorter flash suppression durations caused a dramatic decrease in the amount of successfully triggered traveling waves. The model explains this in terms of the slow adaptation to the dominant percept, which has to reach a certain level in order for the carrier grating to be able to suppress the mask grating.
